# Long-term survival by surgery and adjuvant chemotherapy in a patient with perforated extranodal NK/T-cell lymphoma of the small intestine: a retrospective case study

**DOI:** 10.1186/s40792-023-01688-7

**Published:** 2023-06-12

**Authors:** Hiroshi Funaki, Naomi Nojima, Yutaka Takikawa, Kazutoshi Komori, Hajime Hasegawa, Tomoyuki Sakai, Sohsuke Yamada, Yasufumi Masaki

**Affiliations:** 1 Department of Surgery, Ushitsu General Hospital, 97 Ta-Aza, Ushitsu, Housu-Gun, Noto-Cho, Ishikawa 927-0495 Japan; 2 Department of Hematology and Immunology, Kanazawa Medical University, Kahoku, Ishikawa Japan; 3 Department of Pathology and Laboratory Medicine, Kanazawa Medical University, Kahoku, Ishikawa Japan

**Keywords:** Extranodal NK/T-cell lymphoma, Nasal type, Small intestine, Perforation, Long-term survival, Adjuvant chemotherapy

## Abstract

**Background:**

Extranodal natural killer/T-cell lymphoma, nasal type (ENKL) of the small intestine, is a disease with extremely poor prognosis. We describe treatment in a case which is novel in that it demonstrated long-term survival.

**Case presentation:**

A 68-year-old man was admitted to the emergency department of our hospital with the complaint of severe umbilical pain with tenderness and muscular defense. An abdominal computed tomography scan revealed a thick-wall mass on the small intestine and intra-abdominal free air. He was suspected of perforation of a small intestinal tumor and underwent emergency surgery. The surgery revealed a perforated tumor ulcer, and ENKL was diagnosed from the postoperative pathological findings. The patient’s postoperative course was uneventful. He was further treated with adjuvant chemotherapy by hematologist comprising six courses of dexamethasone, etoposide, ifosfamide, and carboplatin. The patient demonstrated long-term survival and was in remission at the time of writing, four years and five months after surgery.

**Conclusions:**

We report a rare case of long-term survival of perforated ENKL of the small intestine achieved by surgery and adjuvant chemotherapy with dexamethasone, etoposide, ifosfamide, and carboplatin. It is essential to consult with a hematologist to determine the most appropriate chemotherapy such as DeVIC if one encounters rare postoperative pathological findings of ENKL. To elucidate the pathophysiology of this disease and to prolong survival of affected patients, accumulation of cases of long-term survival and examination of associated characteristics is necessary.

## Background

Extranodal natural killer/T-cell lymphoma, nasal type (ENKL), is a rare variant of malignant lymphoma [1]. Although ENKL comprises 3–10% of all lymphomas in East Asia but less than 1% in Western countries, the clinical features of ENKL are similar worldwide [2, 3]. About 70% of ENKL are found in the nasal cavity or nasopharynx as localized disease; however, its primary tumor can occur outside nasal areas, such as in skin, soft tissue, and the gastrointestinal (GI) tract [3, 4]. ENKL of the GI tract is rare with extremely poor prognosis despite active treatment including chemotherapy and surgery [5–7]. Asia Lymphoma study group reported a median survival of 7.8 months despite active treatment including chemotherapy and surgery [5]. Nakagawa et al*.* reported all cases died within 6 months among which 75% died within 2 months among 12 cases with perforated ENKL of GI tract in Japan [6]. We here present an especially rare case of long-term survival with perforated ENKL of the small intestine achieved by surgery and adjuvant chemotherapy.

## Case presentation

**Fig. 1:**
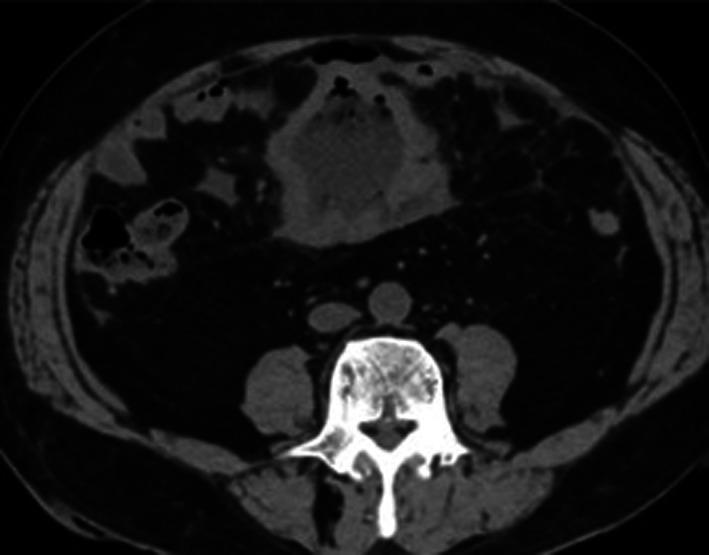
Abdominal computed tomography (CT) scan image before surgery. Abdominal CT scan revealed an 82 × 79 mm thick-wall mass connected to the small intestine and also intra-abdominal free air

**Fig. 2:**
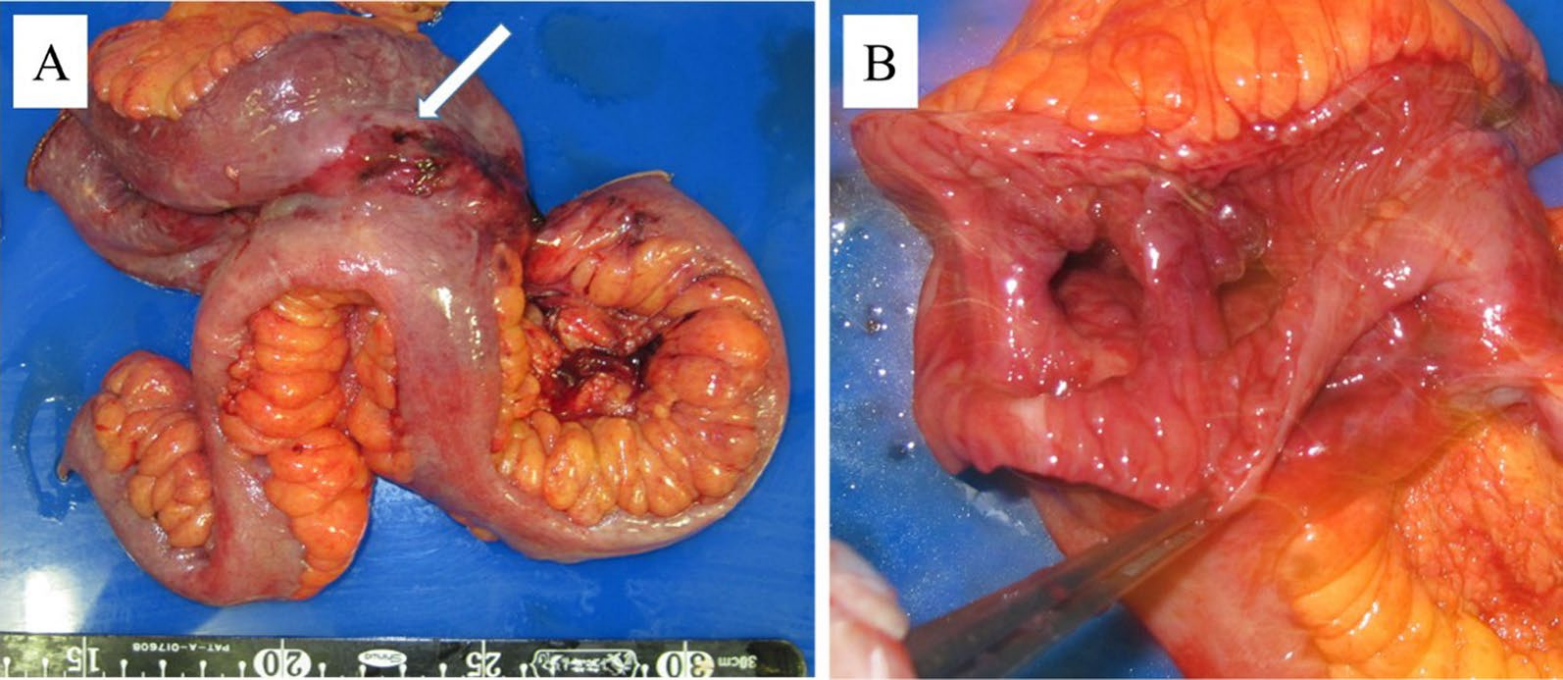
Surgical specimen. A resected specimen showed perforation of the tumor ulcer (**A** arrow), its invasion to nearby the small intestine (**A**), and penetration to the meso-jejunum (**B**)

**Fig. 3:**
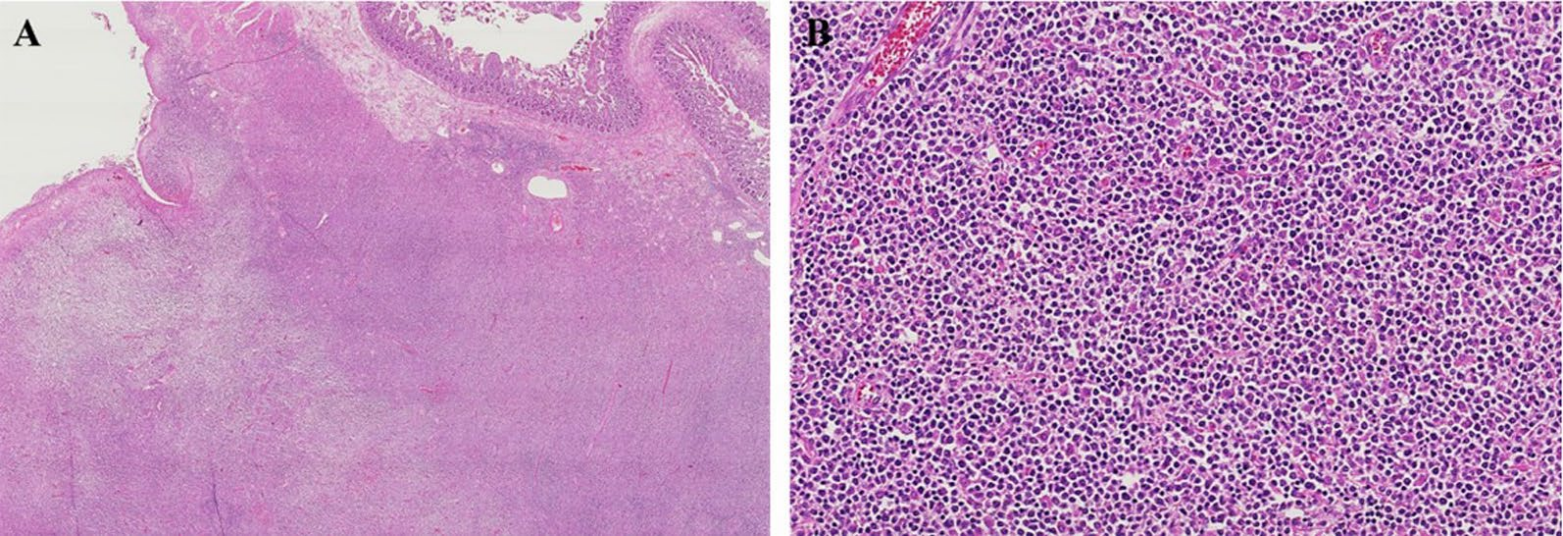
Hematoxylin and eosin staining. **A** Histological examination at low magnification revealed the infiltrative tumor comprised a solid and diffuse proliferation of overtly atypical cells, aggressively involving the jejunal wall especially in the serosa to submucosa, but not in the mucosa. **B** Hemorrhage and necrosis were not so obvious. High magnification showed the atypical lymphoid cells were medium-sized, rounded and hyperchromatic enlarged nuclei, admixed with occasional mitotic figures (8–10/10 high-power fields)

**Fig. 4:**
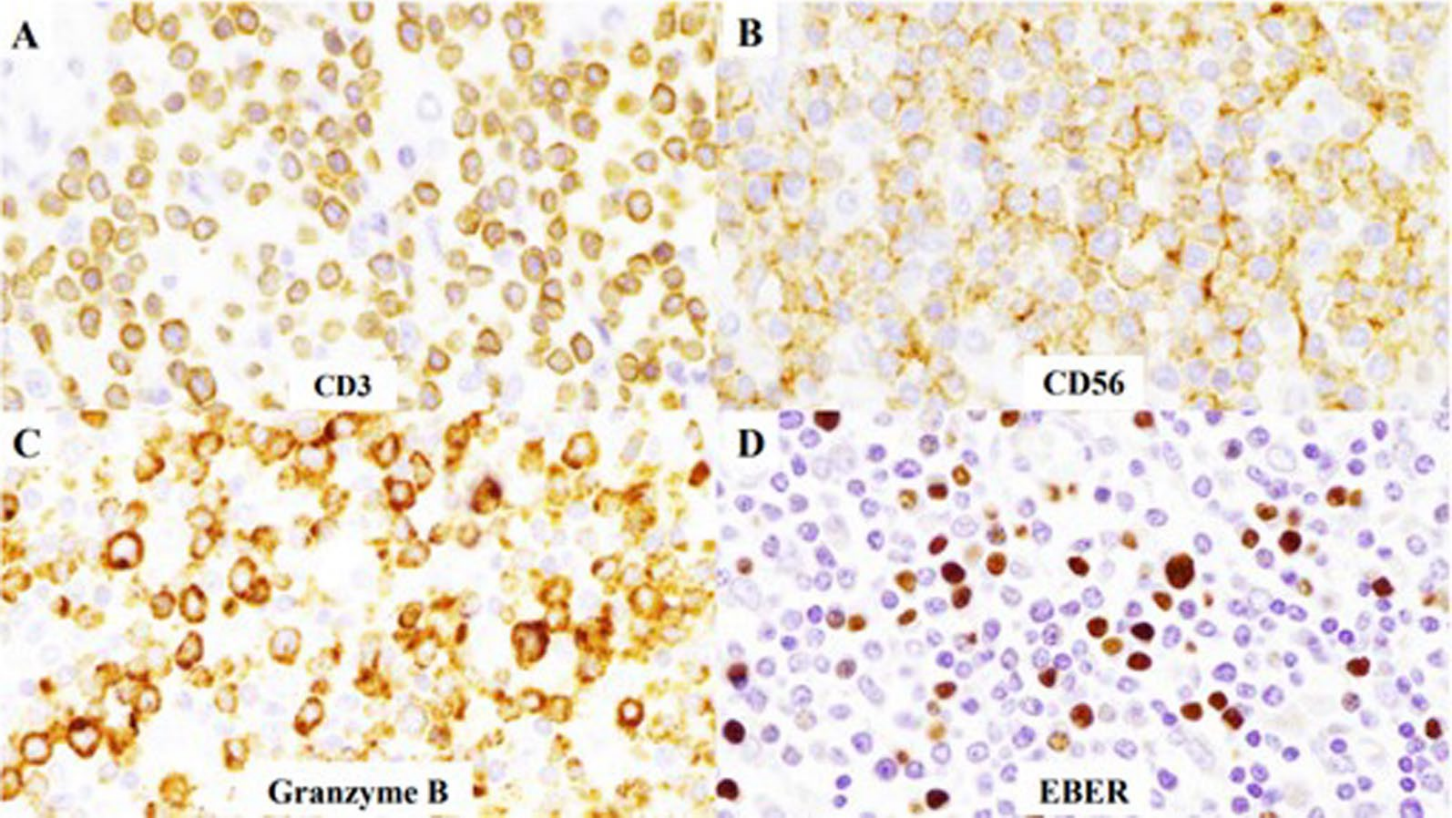
Immunohistochemistry. **A** Positive staining for cytoplasmic CD3. **B** positive staining for CD56. **C** Positive staining for Granzyme B. **D** EBER in situ hybridization stain for EBV was partly positive in these lymphoma cells

A 68-year-old man was admitted to the emergency department of our hospital with the complaint of severe umbilical pain. He had noticed umbilical pain three times within a month. His blood pressure was 153/96 mmHg, heart rate elevated but regular at 101 bpm, and body temperature mildly elevated at 37.1 °C. The peripheral capillary oxygen saturation was 92% at room air. During examination, tenderness and rebound tenderness of the umbilical lesion, and muscular defense were observed. Laboratory tests showed anemia with hemoglobin level 10.9 g/dL and high inflammatory response with C-reactive protein level 6.46 mg/dL. Serum lactate hydrogenase was 162 U/L. A coagulation examination was normal. Abdominal computed tomography (CT) scan revealed an 82 × 79 mm thick-wall mass on the small intestine and intra-abdominal free air (Fig. 1). Suspected with a perforated small intestinal tumor, the patient underwent emergency surgery with small bowel resection and intra-peritoneal drainage. A resected specimen showed perforated tumor ulcer (Fig. 2A, arrow). Invasion of the tumor to the nearby small intestine (Fig. 2A) and an 8 × 5 cm lesion by tumor ulcer penetration to the meso-jejunum (Fig. 2B) was revealed by abdominal CT (Fig. 1). ENKL was diagnosed from postoperative pathological findings. Histological examination at low magnification showed the infiltrative tumor comprised a solid and diffuse proliferation of overtly atypical cells, aggressively involving the jejunal wall especially in the serosa to submucosa, but not in the mucosa (Fig. 3A). Hemorrhage and necrosis were not so obvious. High magnification revealed atypical lymphoid cells as medium-sized, rounded and hyperchromatic with enlarged nuclei, admixed with occasional mitotic figures (8–10/10 high-power fields) (Fig. 3B). In immunohistochemistry, the tumor cells showed positive for CD45, cytoplasmic CD3 (Fig. 4A), CD56 (Fig. 4B) and Granzyme B (Fig. 4C), whereas negative for CD4, CD5, CD8 and CD20. Moreover, the Epstein–Barr virus (EBV)-encoded small RNAs (EBER) in situ hybridization (ISH) stain for EBV was partly positive in these lymphoma cells (Fig. 4D). Taken together, all these features were consistent with diagnosis of malignant lymphoma, ENKL, of the jejunum. The patient’s postoperative course was uneventful, and he was discharged on postoperative day 14. The patient presented to a hematologist in a university hospital promptly after discharge from our hospital and underwent examinations. Bone-marrow aspiration showed no bone-marrow invasion and fluorodeoxyglucose positron emission tomography–computed tomography (FDG–PET/CT) revealed no residual tumor. The adjuvant chemotherapy was started when physical strength was sufficiently recovered at 1-month postoperative, and that six courses of DeVIC [8, 9] (carboplatin, 300 mg/m^2^ on day 1; etoposide, 100 mg/m^2^ on days 1–3; ifosfamide, 1500 mg/m^2^ on days 1–3; and dexamethasone, 33 mg/body on days 1–3) were performed. The patient was followed-up by regular visits to the hospital and remains in remission at 4 years 5 months.

## Discussion

In the 5th edition of World Health Organization Classification (2022), ENKL is classified as NK-cell tumor concomitant with aggressive NK-cell leukemia and chronic lymphoproliferative disorder of NK-cells [1]. Although the majority of ENKL cases originate from NK-cells, it is often difficult to differentiate NK- from T-cells with current techniques [3]. Therefore, both are collectively described as NK/T-cell lymphoma [3]. Though the present case was diagnosed as ENKL by postoperative pathological findings, which is known to be rare disease with extremely poor prognosis, he is showing long-term survival following small bowel resection and adjuvant chemotherapy with DeVIC (dexamethasone, etoposide, ifosfamide, and carboplatin).

Retrospective clinicopathological analysis of patients with ENKL involving the GI tract was reported by Asia Lymphoma study group [5]. Of 81 patients, over 60% presented as advanced disease, and 68% had GI manifestations including abdominal pain, GI tract bleeding and bowel perforation. Although univariate analysis showed extranodal involvement and performance status were significantly associated with overall survival, there was no significant association for age, serum lactate hydrogenase elevation, stage, B symptoms, location of GI tract involvement, nasal involvement, or bone-marrow invasion. Although characteristics directly associated with the aggressive nature of this disease are elusive, patients that underwent surgery plus chemotherapy showed a trend of better survival than those treated with chemotherapy alone [5]. Our case suggests that, although perforated ENKL of the small intestine is advanced, curative resection with no post-surgery complication and prompt induction of adjuvant chemotherapy may facilitate long-term survival.

Pathologically Epstein–Barr virus (EBV) infection has been suggested as a pathogenic factor [10] and ENKL cells express T-cell markers such as CD2, cytoplasmic CD3 (CD3ε), and CD45 as well as NK-cell marker CD56 [11]. Perforin, Fas ligand, and intercellular adhesion molecule-1 are also shown. The ENKL cell was initially thought to be originated from NK-cells alone by reason that the gene rearrangement of T-cell receptors was not found [11]. However, a number of cases with such rearrangement has been reported, indicating that some ENKL are derived from T-cell lineage. The current concept of the origin of ENKL is NK- or γδ T-cells lineage as first proposed by Harabuchi et al. [12]. Our case showed positive for CD45, cytoplasmic CD3 (Fig. 4A), CD56 (Fig. 4B) and Granzyme B (Fig. 4C), yet completely negative for CD4, CD5, CD8 and CD20. Moreover, EBV–ISH was positive in lymphoma cells (Fig. 4D). Taken together, all these features are consistent with malignant lymphoma, ENKL, of the jejunum. For differential diagnosis, although enteropathy-associated T-cell lymphoma was mentioned, our case was diagnosed as ENKL, because the main infiltrating site was in the serosa to submucosa but not the mucosa, and CD56- and EBV–ISH findings were positive.

Therapeutic strategies for malignant tumors are often decided according clinical or pathological staging. Because the main lesion of malignant lymphoma of the GI tract is extranodal, it often diverges from the progression of stage in the Ann Arbor classification [4]. Therefore, for malignant lymphoma of the primary GI tract, the Lugano staging prepared by the International Congress of Malignant Lymphoma is used [13]. In our case, since tumor penetrated to the nearby small intestine, it became Stage IIE by Lugano staging for GI tract lymphoma.

ENKL is an aggressive lymphoma and has been traditionally treated using the same strategy as other aggressive lymphomas, mainly with anthracycline combination chemotherapy: CHOP (cyclophosphamide, doxorubicin, vincristine, and prednisolone) [3]. Tumor cells in ENKL express the multidrug resistance (MDR) gene and its product, P-glycoprotein [3]. This MDR phenomenon is believed to be the major reason why ENKL is resistant to CHOP chemotherapies that consist mainly of MDR-related agents, such as doxorubicin and vincristine, and has led to the development of non-MDR-related chemotherapy [3]. Although treatment varies from country to country because of epidemiological racial differences, in Japan, concurrent chemoradiotherapy consisting of radiotherapy and two-thirds dose of DeVIC (RT-2/3DeVIC) is recommended for first-onset localized ENKL of the nasal cavity, and SMILE therapy (dexamethasone, methotrexate, ifosfamide, l-asparaginase, etoposide), systemic chemotherapy, is recommended for first onset advanced stage and first relapse/refractory ENKL [14]. DeVIC and SMILE regimes are both MDR independent to work drugs [15]. Although our case was in a very advanced condition, we choose DeVIC regimen for adjuvant chemotherapy, because bone-marrow aspiration and FDG–PET showed no residual tumor after surgery.

The present case showed intestinal perforation on admission and underwent systemic chemotherapy after surgery. The role of surgery in GI lymphoma has been a topic of debate. For gastric lymphoma, non-surgical treatment such as chemotherapy has shown equal or better outcomes according to Ferreri et al. [16], based on retrospective and prospective data. Japanese guidelines for malignant lymphoma indicate gastrectomy be performed in cases with complications such as massive bleeding or perforation during chemotherapy and as salvage surgery for non-responders, but Helicobacter pylori eradication or chemotherapy is the preferred first-line treatment [17]. For colorectal lymphoma, Cai et al. reported that surgical intervention did not improve survival in patients with advanced stage, left-sided, or indolent colorectal lymphoma, whereas favorable outcomes were found in those with early tumor stage, right-sided lesion, or diffuse large B-cell histological [18]. Surgery may be reserved for patients with advanced stage GI lymphoma to prevent further complications such as high risk of bleeding or perforation.

## Conclusions

We report an extremely rare case of long-term survival of perforated ENKL of the small intestine, achieved by surgery and adjuvant chemotherapy with DeVIC regimen. It is essential for the surgeon to consult with a hematologist to determine the most appropriate adjuvant chemotherapy such as DeVIC if one encounters rare postoperative pathological findings of ENKL, since there are currently no guidelines or evidence of best practice for postoperative adjuvant chemotherapy for this condition.

## Data Availability

All data are available on request.

## Abbreviations

DeVIC: Carboplatin, etoposide, ifosfamide, dexamethasone
EBER: Epstein–Barr virus-encoded small RNAs
EBV: Epstein–Barr virus
ENKL: Extranodal natural killer/T cell lymphoma, nasal type
GI: Gastrointestinal
MDR: Multidrug resistance
SMILE: Dexamethasone, methotrexate, ifosfamide, l-asparaginase, etoposide
